# Postoperative changes of intermittent exotropia type as classified by 1-hour monocular occlusion

**DOI:** 10.1371/journal.pone.0200592

**Published:** 2018-08-01

**Authors:** Seok Hyun Bae, Young Bok Lee, Soolienah Rhiu, Joo Yeon Lee, Mi Young Choi, Hae Jung Paik, Key Hwan Lim, Dong Gyu Choi

**Affiliations:** 1 Department of Ophthalmology, Kangnam Sacred Heart Hospital, Hallym University College of Medicine, Seoul, Korea; 2 Department of Ophthalmology, Dongtan Sacred Heart Hospital, Hallym University College of Medicine, Dongtan, Korea; 3 Department of Ophthalmology, Hallym University Sacred Heart Hospital, Hallym University College of Medicine, Anyang, Korea; 4 Department of Ophthalmology, Chungbuk National University Hospital, Chungbuk National University College of Medicine, Cheongju, Korea; 5 Department of Ophthalmology, Gachon University Gil Medical Center, Gachon University School of Medicine, Incheon, Korea; 6 Department of Ophthalmology, Ewha Womans University Mokdong Hospital, Ewha Womans University School of Medicine, Seoul, Korea; Faculty of Medicine, Cairo University, EGYPT

## Abstract

**Purpose:**

To evaluate postoperative changes of the intermittent exotropia type as classified by 1-hour monocular occlusion test.

**Design:**

Institutional, retrospective study.

**Methods:**

We retrospectively reviewed the medical records of 179 patients who had undergone surgery for intermittent exotropia with a postoperative follow-up of 6 months or more. We evaluated the exodeviation obtained before and after 1-hour monocular occlusion preoperatively and again at postoperative 1, 3 and 6 months. Intermittent exotropia was divided into 4 types according to Burian’s classification. The main outcome measure was the distribution of intermittent exotropia type based on 1-hour monocular occlusion in both pre- and postoperative periods.

**Results:**

Of the 179 patients, 152 (84.9%) were assigned preoperatively to the basic type, 14 (7.8%) to the pseudo-divergence excess type, and 13 (7.8%) to the convergence insufficiency type. At postoperative 1, 3, and 6 months, the exotropia-type distribution was shifted predominantly to the basic type (p<0.001, p = 0.004, p = 0.029, respectively). Among the preoperative basic-type patients, 96.9% maintained that type postoperatively. However, only 18.2 and 11.1% of the pseudo-divergence excess and convergence insufficiency types maintained the same type. The proportions of the basic type had increased at postoperative 6 months, from 87.8 to 95.7% for bilateral lateral rectus (BLR) recession, from 73.7 to 92.3% for unilateral recess-resect (R&R), and from 88.0 to 95.0% for unilateral lateral rectus (ULR) recession.

**Conclusion:**

The type of intermittent exotropia changed mostly to the basic type postoperatively even as classified after 1-hour monocular occlusion. This finding was consistent regardless of the surgical methods (BLR, ULR recession and R&R).

## Introduction

Intermittent exotropia is the most common type of strabismus in East Asia (i.e., Korea, Japan and China) [1–4]. Burian et al., having classified exotropia based on differences of distance and near deviation, recommended different surgical methods for each of the following four types: (1) the basic type, defined as exodeviation that varies only within physiologic limits in distance and near fixation; (2) the true divergence excess type, defined as exodeviation at distance 10 prism diopters (PD) larger than at near fixation, with no post-occlusion change in the deviation at near; (3) the pseudo-divergence excess type, defined as exodeviation at distance 10 PD larger than at near fixation, but with increased near deviation within 10 PD when monocular occlusion is applied; (4) the convergence insufficiency type, defined as greater, 10 PD or more exodeviation at near than at distance [5–7]. They classified exodeviations by near measurement according to both short-duration monocular occlusion and the addition of +3.00 diopter (D) spheres over each eye. Occluding one eye serves to exclude fusional stimuli, and adding +3.00 D spheres removes the effect of accommodative convergence [5–7]. Kushner and Richard have suggested that this can be attributed to a strong fusional mechanism defined as “tenacious proximal fusion” [8]. Several previous studies have found that 1-hour occlusion was sufficient for clinical application [7, 9, 10]. Burian and Franceschetti found the basic type to be the most common exotropia (49%) and the true divergence excess type the least common (6%) [6].

Most clinicians, in choosing their surgical methods, have considered Burian’s classifications based on the preoperative 1-hour monocular occlusion test. Burian et al. classically suggested bilateral lateral rectus recessions for the divergence excess type and a recess-resect for the basic type and pseudo-divergence excess type, according to the unproven hypothesis that bilateral lateral rectus recessions would affect the distance deviation more than the near deviation and that a recess-resect would affect the distance and near angles equally [5–7, 10]. Successful motor outcome after the surgery has been attributed to the decrease of distance and near angles resulting in the collapse of distance-near discrepancy. So many studies have reported surgical results for intermittent exotropia with postoperative deviation angles at both near and distance fixation [8, 10–13]. However, the true divergence excess type was not differentiated from the pseudo-divergence excess type postoperatively, because the occlusion test was not performed for recurrent exotropia patients [12].

To the best of our knowledge, there has been no study on the use of the 1-hour diagnostic monocular occlusion test for postoperative intermittent exotropia classification. Therefore, we undertook the present study in order to classify exotropia into 4 types postoperatively as well as preoperatively based on the 1-hour monocular occlusion test and to evaluate the postoperative changes of exotropia types. Consequently, we tried to investigate the possible mechanism of recurrent or residual exodeviation postoperatively.

## Materials and methods

A retrospective medical records review was conducted for 179 consecutive patients who had undergone surgery for intermittent exotropia and for whom pre- and post-1-hour-occlusion deviation-angle measurement data were available. The minimum required postoperative follow-up period was 6 months. Patients having a history of previous strabismus surgery or infantile exotropia, sensory exotropia, paralytic exotropia, restrictive exotropia, or neurologic disorder such as Down syndrome or cerebral palsy had been excluded, as had those who had undergone oblique or vertical rectus muscle surgery combined with exotropia surgery. This study was approved by the Institutional Review Board of the Hallym University Medical Center (IRB No. 2015-03-33).

### Preoperative examinations

We reviewed the patients’ preoperative characteristics including age at surgery, sex, preoperative deviation at distance and near, best-corrected visual acuity (BCVA), refractive error, presence of lateral gaze incomitance, amblyopia, dissociated vertical deviation (DVD), and vertical deviation. Lateral gaze incomitance was defined as 20% or more change of angle deviation relative to the primary position. Amblyopia was defined as a visual-acuity difference of 2 or more lines. Vertical deviation was defined as hypertropia/hypotropia of 5 PD or more in the primary position.

All of the patients underwent a complete ophthalmic examination preoperatively. Preoperative deviation was determined by alternate prism cover test at distance (6m) and near (0.33m). Additional distance and near measurements were obtained after 1-hour monocular occlusion of either eye. If, after 1-hour occlusion, exodeviation remained 10 PD larger at distance than at near fixation, one final post-occlusion measurement at near was obtained with a +3.00 D sphere over each eye before allowing the patient to regain binocular fusion. Refractive error was measured by streak retinoscopy after cycloplegia using 1% cyclopentolate hydrochloride and 1% tropicamide. Patients with amblyopia underwent part-time occlusion along with best-corrected spectacles wearing before the operation. No patient had taken overminus lens therapy.

### Surgery

All of the patients underwent, under general anesthesia, exotropia surgery by 6 surgeons at 6 institutions. Bilateral lateral rectus (BLR) recession, unilateral recess-resect (R&R), or unilateral lateral rectus (ULR) recession was performed. The selection between BLR recession and R&R was made by the operating surgeon according to his/her preference. ULR recession could be performed for cases of exotropia less than 25 PD. Some patients simultaneously underwent supra-/infra-transposition of the horizontal muscle to correct small vertical deviation or A-V pattern. Surgical dosages were based on the largest deviation for distance as measured after 1-hour monocular occlusion, as indicated in Table 1.

**Table 1:** Surgical dosages for intermittent exotropia patients.

PD	BLR (mm)	R&R (mm)	ULR (mm)
**15**	4.0	4.0/3.0	8.0
**20**	5.0	5.0/4.0	9.0
**25**	6.0	6.0/5.0	10.0
**30**	7.0	7.0/5.5	
**35**	7.5	7.5/6.0	
**40**	8.0	8.0/6.5	
**50**	9.0	9.0/7.0	

PD, prism diopters; BLR, bilateral lateral muscle recession; R&R, unilateral lateral rectus recess-medical rectus resect; ULR, unilateral lateral rectus recession.

### Postoperative examination and management

The alignment at distance and near was measured by alternate prism cover test at postoperative 1, 3 and 6 months. In every case, the measurements were made before and after 1-hour occlusion of one eye. As in the preoperative examination, when the exodeviation of 10 PD or more remained after 1-hour occlusion, near deviation was obtained with a +3.00 D sphere over each eye before allowing the patient to regain binocular fusion.

Alternate full-time patching was prescribed and continued until disappearance of the postoperative diplopia or esodeviation. In cases of reoperation due to recurrence of exotropia or consecutive esotropia, the data prior to second operation were included in the analysis.

### Main and secondary outcome measures

The main outcome measure was the exotropia-type distributions obtained before and after 1-hour monocular occlusion in the pre- and postoperative periods. We also analyzed the postoperative changes of the exotropia types by preoperative type and surgical procedure.

The definitions of the types of exotropia pre- and postoperatively are as follows: (1) basic type: exodeviation within 10 PD difference between at distance and near; (2) true divergence excess type: exodeviation at distance ≥ 10 PD larger than at near, even after monocular occlusion; (3) pseudo-divergence excess type: exodeviation at distance ≥ 10 PD larger than at near, but with increased near deviation within 10 PD after monocular occlusion; (4) convergence insufficiency type: near deviation ≥ 10 PD greater than distance deviation.

The secondary outcome measure was the surgical success rates and postoperative changes of the distribution of the exotropia type in patients with overcorrection or undercorrection. Surgical success was defined as an alignment between exodeviation of 10 PD and esodeviation of 5 PD at distance and near at postoperative 6 months. Overcorrection was defined as esodeviation of 5 PD or more, and undercorrection as exodeviation of 10 PD or more.

### Statistical analysis

The statistical analysis was performed with SPSS software for Windows (version 23.0, SPSS Inc., Chicago, Illinois, USA). The Pearson chi-square test was used for the comparison of the type of intermittent exotropia between the preoperative and postoperative periods. A probability value less than .05 was considered statistically significant. In the patients who had achieved orthotropia at distance and near postoperatively, the basic type was considered in the analysis.

## Results

### Preoperative demographic data

A total of 179 patients (94 males, 85 females) were enrolled. Their demographic data are shown in Table 2. The mean age at surgery was 9.2 ± 6.6 years (range 3–58 years). The mean pre-occlusion exodeviations were 26.9 ± 8.0 PD at distance and 26.8 ± 10.8 PD at near. After 1-hour monocular occlusion, the mean exodeviations were 27.6 ± 7.8 PD and 30.4 ± 9.0 PD at distance and at near, respectively; thus, the exodeviations had increased 0.61 ± 2.53 PD at distance and 3.72 ± 6.34 PD at near.

**Table 2:** Preoperative demographic data.

Variables	Total (n = 179)
**Age at surgery (years)**	9.2 ± 6.6
**Sex (Male/Female)**	94/85
**Preoperative angle of exodeviation (PD)**	
at distance (pre-occlusion)	26.9 ± 8.0
at near (pre-occlusion)	26.8 ± 10.8
at distance (post-occlusion)	27.6 ± 7.8
at near (post-occlusion)	30.4 ± 9.0
**BCVA (logMAR)**	
Dominant eye	0.03 ± 0.10
Non-dominant eye	0.06 ± 0.15
**Refractive error (D)**	
Dominant eye	-0.53 ± 1.90
Non-dominant eye	-0.65 ± 2.24
**Lateral incomitance (n, %)**	18 (10.1%)
**Amblyopia (n, %)**	10 (5.6%)
**Associated strabismus**	
Dissociated vertical deviation (n, %)	8 (4.5%)
Vertical deviation (n, %)	34 (19.0%)
Oblique muscle dysfunction (n, %)	28 (15.6%)

PD, prism diopters; BCVA, best-corrected visual acuity; D, diopters.

Lateral incomitance: change of 20% or more in lateral gaze from primary position.

Vertical deviation: 5 PD or more hypertropia/hypotropia at primary position.

For the basic type, BLR recession was performed in 67.1% of cases, R&R in 18.4%, and ULR recession in 14.5%. For the pseudo-divergence excess type, the corresponding figures were 71.4, 21.4, and 7.1%, and for the convergence insufficiency type, 30.8, 53.8, and 15.4%, respectively (Table 3).

**Table 3:** Surgical procedures by type of exotropia.

	BLR (n = 116)	R&R (n = 38)	ULR (n = 25)
**Basic type (n = 152)**	102 (67.1%)	28 (18.4%)	22 (14.5%)
**Pseudo-D.E. type (n = 14)**	10 (71.4%)	3 (21.4%)	1 (7.1%)
**C.I. type (n = 13)**	4 (30.8%)	7 (53.8%)	2 (15.4%)

BLR, bilateral lateral muscle recession; R&R, unilateral lateral rectus recess-medical rectus resect; ULR, unilateral lateral rectus recession; Pseudo-D.E., pseudo-divergence excess; C.I., convergence insufficiency.

### Distribution of exotropia type in the pre- and postoperative periods

Table 4 provides the exotropia-type distributions in the pre- and postoperative periods. Preoperatively, of the 179 patients, 152 (84.9%) showed the basic type, 14 (7.8%) the pseudo-divergence excess type, and 13 (7.8%) the convergence insufficiency type and no true divergence excess type. At postoperative 1 month, 97.2% was in the basic type, 1.7% the pseudo-divergence excess type, and 1.1% the convergence insufficiency type, which were significant changes (p<0.001, Pearson chi-square test). At postoperative 3 and 6 months, the distributions were 95.5, 1.3 and 3.2% (p = 0.004) and 94.9, 2.6, and 2.6%, respectively (p = 0.029).

**Table 4:** Changes of distributions of types of exotropia pre- to postoperatively (n = 179).

	Basic	Pseudo-D.E.	True-D.E.	C.I.	P-value*
**Preoperative**	84.9%	7.8%	0%	7.8%	
**Postoperative**					
**1 month**	97.2%	1.7%	0%	1.1%	<0.001
**3 months**	95.5%	1.3%	0%	3.2%	0.004
**6 months**	94.9%	2.6%	0%	2.6%	0.029

Pseudo-D.E., pseudo-divergence excess; True-D.E., true divergence excess; C.I., convergence insufficiency.

*Chi-square test: comparison of type of exotropia between preoperative and postoperative periods.

### Postoperative changes in type of intermittent exotropia by preoperative type

**Fig 1:**
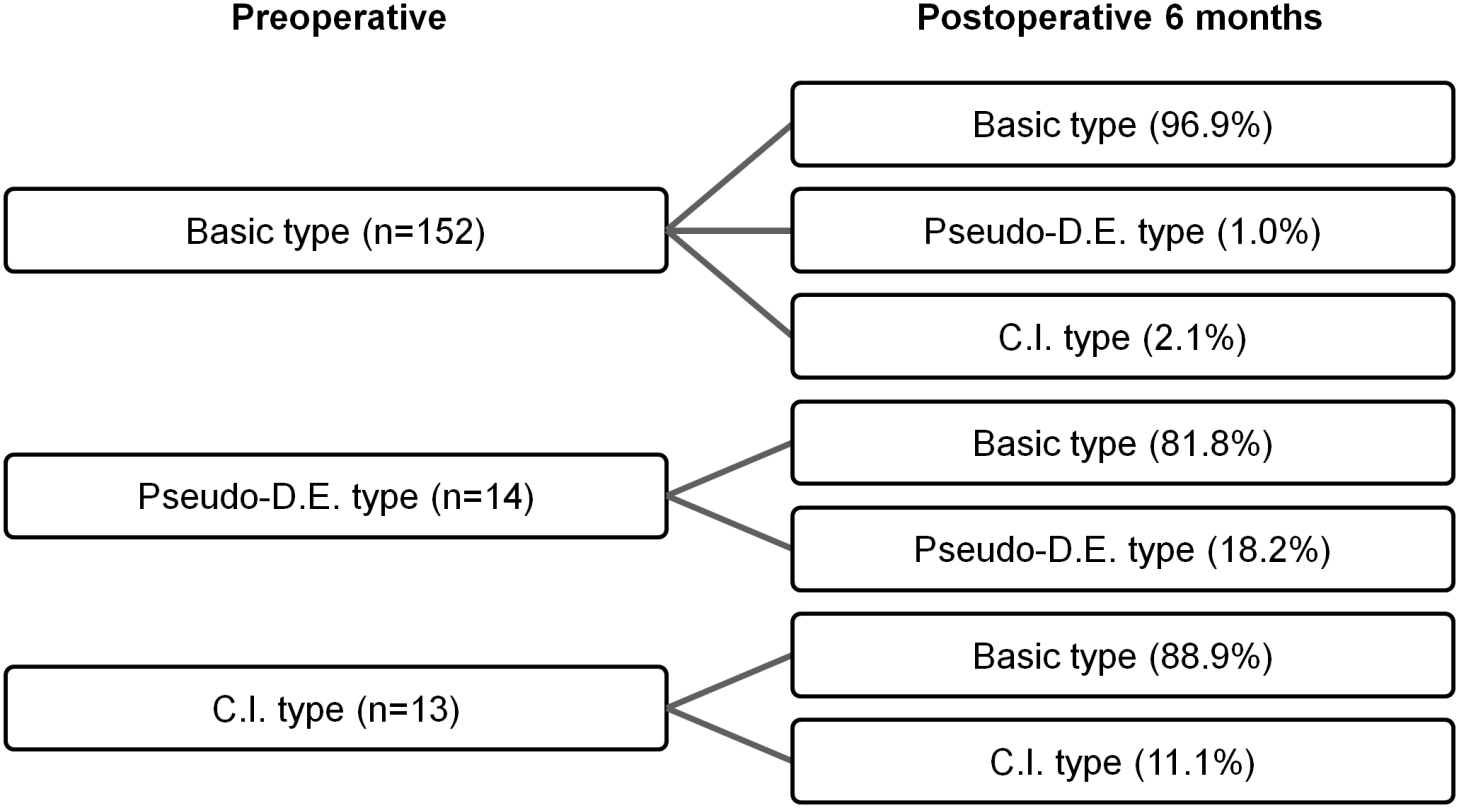
Postoperative distribution of intermittent exotropia types by preoperative type. Pseudo-D.E., pseudo-divergence excess; C.I., convergence insufficiency.

Of the basic type patients, 96.9% maintained that type, while 1% changed to the pseudo-divergence excess type and 2.1% to the convergence insufficiency type. By contrast, most of the pseudo-divergence excess and convergence insufficiency types converted to the basic type; respectively, only 18.2 and 11.1% maintained the same type (Fig 1).

### Postoperative changes in the distribution of exotropia type in patients with overcorrection or undercorrection

Surgical success was achieved in 123 of 179 patients (68.7%) at postoperative 6 months. Overcorrection occurred in 10 patients (5.6%) and undercorrection in 46 (25.7%). Table 5 provides the distributions of exotropia types at pre- and postoperative 6 months in patients with overcorrection or undercorrection. Among patients with overcorrection, 80.0% were in the basic type, 10.0% the pseudo-divergence excess type, and 10.0% the convergence insufficiency type preoperatively. At postoperative 6 months, 80.0% were in the basic type, 20.0% the pseudo-divergence excess type, and no convergence insufficiency type. Among the patients with undercorrection, the distributions changed from 82.6, 4.4 and 13.0% preoperatively to 91.3, 2.2 and 6.5% at postoperative 6 months, respectively, in which the proportion of the basic type increased postoperatively in patients with undercorrection. However, statistical analysis was not performed because of a too-small number of patients in each group.

**Table 5:** Postoperative changes in the distribution of exotropia type in patients with overcorrection (n = 10) or undercorrection (n = 46).

	Basic	Pseudo-D.E.	True-D.E.	C.I.
**Overcorrection (n = 10)**				
**Preop. (n, %)**	8(80.0%)	1(10.0%)	0%	1(10.0%)
**Postop. 6 months (n, %)**	8(80.0%)	2(20.0%)	0%	0%
**Undercorrection (n = 46)**				
**Preop. (n, %)**	38(82.6%)	2(4.4%)	0%	6(13.0%)
**Postop. 6 months (n, %)**	42(91.3%)	1(2.2%)	0%	3(6.5%)

Overcorrection = esotropia ≥ 5 prism diopters (PD) at postoperative 6 months

Undercorrection = exotropia ≥ 10 PD

Pseudo-D.E., pseudo-divergence excess; True-D.E., true divergence excess; C.I., convergence insufficiency.

### Changes of exotropia type according to surgical procedure

**Fig 2:**
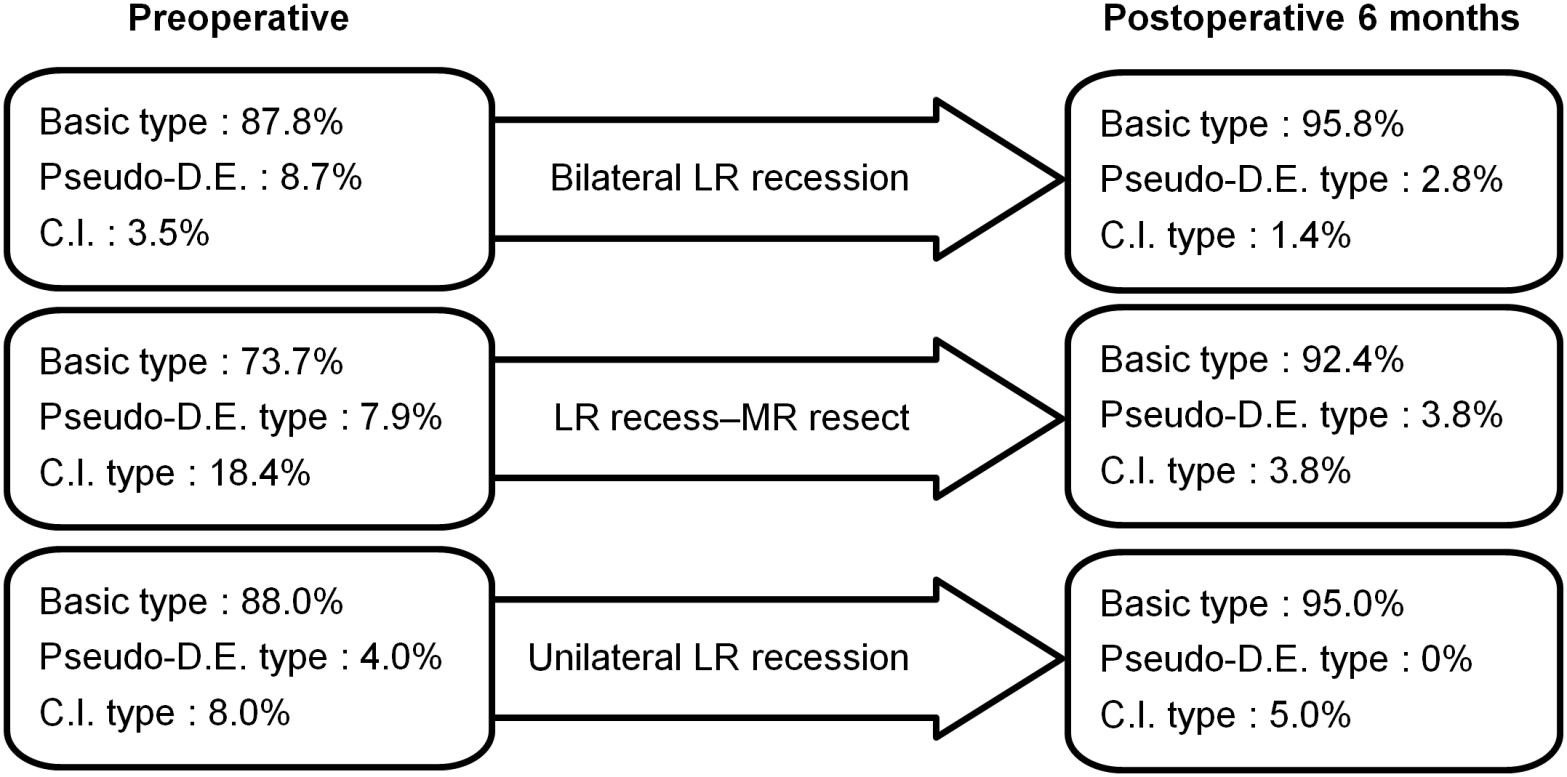
Exotropia distributions by surgical procedure preoperatively and at postoperative 6 months. Pseudo-D.E., pseudo-divergence excess; C.I., convergence insufficiency; LR, lateral rectus; MR, medial rectus.

By postoperative 6 months follow-up, the proportion of the basic type postoperatively had increased compared to that preoperatively: from 87.8% to 95.8% in BLR recession, from 73.7 to 92.4 in R&R, and from 88.0 to 95.0 in ULR recession. Meanwhile, the proportion of the pseudo-divergence excess type had decreased from 8.7% to 2.9%, from 7.9 to 3.8, and from 8.0 to 5.0, respectively, and that of the convergence insufficiency type, from 3.5 to 1.4, from 18.4 to 3.8, and from 8.0 to 5.0, respectively (Fig 2).

## Discussion

Exodeviation classifications were established by Duane, Burian and Kushner to enable physicians to apply the most appropriate surgical method for each type [5, 10, 14]. Deviation measurement after patching of one eye has been performed to determine maximal preoperative exodeviation. Burian recommended short-duration monocular occlusion for differentiating between the true divergence excess and pseudo-divergence excess types [6]. In fact, the one-hour occlusion test has been known to be effective for elimination of fusional impulses [7, 9, 15].

Cho et al. compared exotropia-type distributions between primary and recurrent exotropia according to the distance-near difference after BLR recession. However, they had not performed 1-hour monocular occlusion to differentiate, postoperatively, the pseudo-divergence excess type from the true divergence excess type [12]. As far as we know, our study is the first to have attempted to classify postoperative exotropia types by 1-hour monocular occlusion. In our results, the most frequent preoperative exotropia type was the basic (84.9%), followed by pseudo-divergence excess (7.8%) and convergence insufficiency (7.8%), which data are similar to the findings of previous studies [4, 6, 14]. The basic type maintained itself postoperatively in most cases, and most of the pseudo-divergence excess- and convergence insufficiency-type cases changed to the basic type even after eliminating fusional impulses by 1-hour monocular occlusion test.

We cannot explain the precise underlying mechanism for this result, in which the type of intermittent exotropia changed mostly to the basic type postoperatively. However, Kushner suggested that if distance-near differences in patients with intermittent exotropia were primarily a function of the tenacious proximal fusion mechanism, the relative effect of surgery on the distance and near deviation, respectively, was self-adjusting in patients with basic exotropia, and that the amount of correction obtained at each distance was a function of how much deviation was present [10]. As Kushner supposed, the self-adjusting mechanism in patients who had distance-near differences might be one of the possible explanations for our results. However, proof of this hypothesis requires additional, prospective comparative analysis.

Before this study, we were concerned about the possibility of the secondary convergence insufficiency type after BLR or ULR recession. It might be attributable to an unproven hypothesis that lateral rectus recessions would be more effective at distance than at near fixation, whereas the R&R procedure is equally effective at distance and at near [5, 7, 8, 11]. In fact, Cho et al. reported that the exotropia types in cases of recurrent exotropia after BLR recession showed increasing proportions of the convergence insufficiency type [12]. In the present study, however, the finding in which the type of intermittent exotropia changed mostly to the basic type postoperatively was consistent regardless of the surgical methods (BLR, ULR recession and R&R), and among patients with the basic type preoperatively, the type was changed to convergence insufficiency in only one patient after ULR recession and in just one patient after BLR recession. In the article by Cho et al., the reference for near distance disparity was considered to be 10 PD or more for the patients with distance deviations more than 30PD. However, in the patients with a distance deviation less than 30 PD, the reference value was one-third the distance deviating angle, which might account for the larger number of patients who were classified as convergence insufficiency in the postoperative examinations in their study, since postoperative deviations are generally small. Wang et al. reported that all unilateral medial rectus, bilateral medial rectus recession and improved R&R procedures could reduce near-distance differences in patients with the convergence insufficiency type [13], which results are similar to those of the present study. Moreover, Kushner found in his study that R&R and symmetric BLR recession affected distance-near differences equally in patients with basic exotropia [10]. From this result, he concluded that the hypothesis that lateral rectus recessions selectively affect the distance deviation more than the near deviation, and that the R&R affects both deviations equally, was untrue [10].

There are some limitations to our study. First, the data having been collected at 6 institutions, the surgical procedures differed according to the surgeon’s preference; thus, selection bias could have occurred. Second, no patient with the true divergence excess type or a high AC/A ratio participated in our study, even though those types had not been arbitrarily excluded. Therefore, a larger and randomized prospective study that includes the true divergence excess type should be conducted over a longer period.

In conclusion, the key finding of this study was that most cases of intermittent exotropia changed to the basic type pre-to-postoperatively, even when classifying after 1-hour monocular occlusion. Fortunately, the secondary convergence insufficiency type was only rarely seen after either BLR or ULR recession. When planning the surgery for exotropia in the future, the trend of postoperative changes will be predictable, and this might help reduce the recurrence rate after the surgery.

## Supporting information

S1 File S1_data.xlsx. (XLSX)

## References

[1] Pan CW, Zhu H, Yu JJ, Ding H, Bai J, Chen J, et al Epidemiology of intermittent exotropia in preschool children in china. Optom Vis Sci. 2016;93: 57–62. 26583796 10.1097/OPX.0000000000000754

[2] Chia A, Seenyen L, Long QB. A retrospective review of 287 consecutive children in singapore presenting with intermittent exotropia. J AAPOS. 2005;9: 257–263. 10.1016/j.jaapos.2005.01.007 15956946

[3] Matsuo T, Matsuo C. The prevalence of strabismus and amblyopia in Japanese elementary school children. Ophthalmic Epidemiol. 2005;12: 31–36. 10.1080/09286580490907805 15848918

[4] Oh SY, Huh D, Hwang JM, Min B. The clinical characteristics of intermittent exotropia and their relationship. J Korean Ophthalmol Soc. 1998;39: 2797–2809.

[5] Burian HM. Exodeviation: Their classification, diagnosis, and treatment. Am J Ophthalmol. 1966;62: 1161–1166. 5957892 10.1016/0002-9394(66)92570-0

[6] Burian HM, Franceschetti AT. Evaluation of diagnositic methods for the classification of exodeviations. Trans Am Ophthalmol Soc. 1970;68: 56–71. 5524217 PMC1310365

[7] Burian HM, Spivey BE. The surgical management of exodeviations. Trans Am Ophthalmol Soc. 1964;62: 276–306. 14269896 PMC1310162

[8] Kushner BJ, Morton GV. Distance/near differences in intermittent exotropia. Arch Ophthalmol. 1998;116: 478–486. 9565045 10.1001/archopht.116.4.478

[9] Gürlü VP, Erda N. Diagnostic occlusion test in intermittent exotropia. J AAPOS. 2008;12: 504–506. 10.1016/j.jaapos.2008.02.013 18929306

[10] Kushner BJ. Selective surgery for intermittent exotropia based on distance/near differences. Arch Ophthalmol. 1998; 116: 324–328. 9514485 10.1001/archopht.116.3.324

[11] Kraft SP, Levin AV, Enzenauer RW. Unilateral surgery for exotropia with convergence weakness. J Pediatr Ophthalmol Strabismus. 1995;32: 183–187. 7636700 10.3928/0191-3913-19950501-12

[12] Cho KH, Kim HW, Choi DG, Lee JY. Type of the recurrent exotropia after bilateral rectus recession for intermittent exotropia. BMC Ophthalmol. 2016;16: 97 10.1186/s12886-016-0270-9 27391365 PMC4938985

[13] Wang B, Wang L, Wang Q, Ren M. Comparison of different surgery procedures for convergence insufficiency-type intermittent exotropia in children. Br J Ophthalmol. 2014;98: 1409–1413. 10.1136/bjophthalmol-2013-304442 24842862

[14] Duane A. A new classification of the motor anomalies of the eyes based upon physiological principles, together with their symptoms, diagnosis and treatment. New York: J.H. Vail & Co; 1897.

[15] Kushner BJ. Exotropic deviations: A functional classification and apporach to treatment. Am Orthopt J. 1988; 38: 81–93.

